# Chemical Shift-Encoded Sequence (IDEAL-IQ) and Amide Proton Transfer (APT) MRI for Prediction of Histopathological Factors of Rectal Cancer

**DOI:** 10.3390/bioengineering10060720

**Published:** 2023-06-14

**Authors:** Yang Peng, Xianlun Zou, Gen Chen, Xuemei Hu, Yaqi Shen, Daoyu Hu, Zhen Li

**Affiliations:** Department of Radiology, Tongji Hospital, Tongji Medical College, Huazhong University of Science and Technology, 1095 Jiefang Avenue, Wuhan 430030, China; peterpengyang@126.com (Y.P.); zouxianlun2010@163.com (X.Z.); genchen@hust.edu.cn (G.C.); mayjuly3720@163.com (X.H.); yqshen@hust.edu.cn (Y.S.); daoyuhu@hust.edu.cn (D.H.)

**Keywords:** magnetic resonance imaging, rectal neoplasm, adipose tissue, amides

## Abstract

To investigate whether parameters from IDEAL-IQ/amide proton transfer MRI (APTWI) could help predict histopathological factors of rectal cancer. Preoperative IDEAL-IQ and APTWI sequences of 67 patients with rectal cancer were retrospectively analyzed. The intra-tumoral proton density fat fraction (PDFF), R2* and magnetization transfer ratio asymmetry (MTRasym (3.5 ppm)) were measured according to the histopathological factors of rectal cancer. The relationship between MR parameters and histopathological factors were analyzed, along with diagnostic performance of MR parameters. PDFF, R2* and MTRasym (3.5 ppm) were statistically different between T1+T2/T3+T4 stages, non-metastatic/metastatic lymph nodes, lower/higher tumor grade and negative/positive status of MRF and EMVI (*p* < 0.001 for PDFF, *p* = 0.000–0.015 for R2* and *p* = 0.000–0.006 for MTRasym (3.5 ppm)). There were positive correlations between the above parameters and the histopathological features of rectal cancer (r = 0.464–0.723 for PDFF (*p* < 0.001), 0.299–0.651 for R2* (*p* = 0.000–0.014), and 0.337–0.667 for MTRasym (3.5 ppm) (*p* = 0.000–0.005)). MTRasym (3.5 ppm) correlated moderately and mildly with PDFF (r = 0.563, *p* < 0.001) and R2* (r = 0.335, *p* = 0.006), respectively. PDFF provided a significantly higher diagnostic ability than MTRasym (3.5 ppm) for distinguishing metastatic from non-metastatic lymph nodes (z = 2.407, *p* = 0.0161). No significant differences were found in MR parameters for distinguishing other histopathological features (*p* > 0.05). IDEAL-IQ and APTWI were associated with histopathological factors of rectal cancer, and might serve as non-invasive biomarkers for characterizing rectal cancer.

## 1. Introduction

Colorectal cancer, a common alimentary tumor, accounts for 30–35% of cancer-related death [1]. The surgical operation of total mesorectal excision is applicable for patients at the early stage, while the neoadjuvant chemo-radiotherapy is usually considered for patients with locally advanced rectal cancer [2]. The pathological features of rectal cancer could help decide the selection of surgical operation or neoadjuvant chemo-radiotherapy for its treatment. Previous studies [3,4] reported that patients with rectal cancer of higher T stage, lymph node involvement, poor differentiation and extramural venous invasion (EMVI) have found to be related to local recurrence and distant metastasis. Hence, it is valuable to make an accurate preoperative assessment of histopathological features of rectal cancer, which might facilitate treatment strategies and improve long-term survival rate of patients.

Previous studies [5,6] demonstrated that the adipose tissue, adipocytes and pre-adipocytes in the culture medium could promote the proliferation of colon cancer cells. The adipose tissue owns the characteristics similar to an endocrine organ [7] and influences tumor tissues/cells via cytokines and adipokines [8], which further contribute to cell proliferation and tumor development [9]. Besides, a neighborhood relation is easily detected between the rectum and surrounding meso-rectum with rich fat tissue [10]. Therefore, we hypothesized that the intra-tumoral fat might play an important role in tumor biological behaviors, which were closely associated histopathological features of rectal cancer, based on the relationship between the rectal tumor and adipose tissue.

Non-invasive MRI techniques are utilized to quantify the fat content in different organs and tissues. Magnetic resonance spectroscopy is able to measure fat amount, but it is limited to clinical practice because of a time-consuming scanning process [11]. The iterative decomposition of water and fat with echo asymmetry and least squares estimation (IDEAL-IQ), is available for quantifying fat concentration of rectal tumors by correction of effects caused by T2* and B0 field inhomogeneity [12]. On the other hand, the R2* parameter has been previously investigated to reflect tumor hypoxia [13] and iron deposition [14]. Previous studies [15,16] indicated that R2* value was in proportion to the concentration of deoxyhemoglobin and hypoxia status. This was a first attempt to apply the IDEAL-IQ technique to quantify both intra-tumoral fat and R2* of rectal cancer.

Amide proton transfer-weighted MRI (APTWI) is a relatively novel molecular contrast imaging technique and it could be used for detecting and quantifying endogenous cytoplasmic protein without utilizing exogenous contrast agent [17,18,19]. APTWI is achieved by detection of the chemical exchange rate between amide proton and bulk water [20]. Higher cellular proliferation activity and protein synthesis are noted in malignant tumors, as compared to normal tissues and benign tumors, so this difference of cellularity and proteins could be imaged by APTWI. It has been performed in many cancers, such as prostate cancer [21], cervical cancer [22], breast cancer [23] and lung cancer [24]. Even though a few studies [17,19] have demonstrated the relationship between APTWI and histopathological prognostic factors of rectal cancer, and its role in the treatment efficacy of rectal cancer [25,26], no studies investigated APTWI combined with IDEAL-IQ in assessment of histopathological features of rectal cancer.

Therefore, with respect to the available MR data, our study aimed to determine the relationship between IDEAL-IQ/APTWI-derived parameters and the histopathological factors of rectal cancer to provide new ideas for diagnosis and treatment of rectal cancer.

## 2. Materials and Methods

### 2.1. Study Subjects

This retrospective study was sanctioned by the institutional review board of our hospital. The written informed consent was waived for all patients prior to their enrollment in this investigation. Between April 2016 and May 2019, 108 patients with suspicious rectal tumors were selected for rectum MRI imaging including both IDEAL-IQ and APTWI sequences. The inclusion criteria were as follows: (1) pathologically confirmed rectal cancer by surgery; (2) acceptable image quality of IDEAL-IQ and APTWI for measurement; (3) no previous chemo-radiotherapy or surgical operations before MR scanning; (4) time interval between biopsy examination and MR scanning surpassed four days.

**Figure 1:**
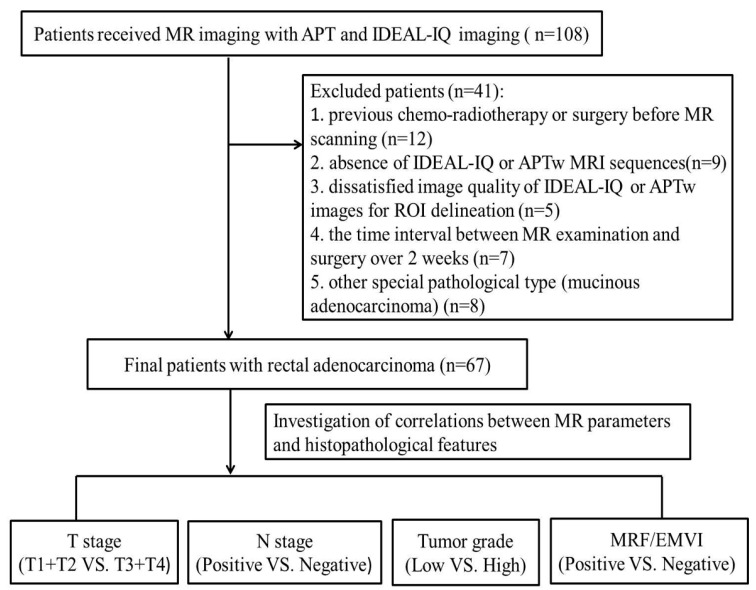
Flow diagram for selection process of rectal cancer patients.

As a result, 41 patients were excluded because of the following factors: (1) previous chemo-radiotherapy or surgery before MR scanning (12 patients); (2) absence of IDEAL-IQ or APTWI sequences (nine patients); (3) dissatisfied image quality of IDEAL-IQ or APTWI images for ROI delineation (five patients); (4) the time interval between MR examination and surgery over 2 weeks (seven patients); (5) other special pathological type (mucinous adenocarcinoma) (eight patients) (Figure 1). 

### 2.2. MRscanning Protocols

A 3.0T MR scanner (Discovery MR750, GE Healthcare, Waukesha, WI, USA) was applied for rectal cancer patients. A 32-channel phased-array coil was utilized in the scanning process. Each studied subject was intramuscularly injected with 5 mg racanisodamine hydrochloride 20 min before MR examination to prevent artifacts caused by intestinal peristalsis. Our MR imaging protocol was included as follows: T1-weighted imaging (WI), T2WI, diffusion-weighted imaging (DWI), IDEAL-IQ and APTWI (Table 1). The DWI, IDEAL-IQ and APTWI sequences were scanned vertically to the long axis of rectal lesions by utility of sagittal T2WI as reference.

### 2.3. Image Processing and Analysis

All the APTWI images were transferred to a GE workstation (AW 4.6, GE healthcare, USA), and the corresponding images were processed and analyzed using the APT software in the functional kit. The region of interest (ROI) was contoured by delineating the margin of tumor tissue on the axial APT image using axial high-resolution T2WI and DWI images as reference. Every ROI was placed on the corresponding APT image (single slice) at the largest section of each tumor to cover the solid region of tumor as much as possible. The necrotic, cystic and bleeding areas should be avoided for ROI delineation. 

The APTWI-derived parameter (MTRasym) was calculated by utility of the following equation:

MTRasym (3.5 ppm) = [Ssat (−3.5 ppm) − Ssat (+3.5 ppm)]/S0

(MTRasym (3.5 ppm) refers to the magnetization transfer ratio at 3.5 ppm, S0 indicates the signal intensity of APTw without applying the saturation pulse, and Ssat is the signal intensity of APTw after applying the saturation pulse).

As for the IDEAL-IQ imaging technique, the proton density fat fraction (PDFF) maps and R2* maps were automatically produced by the corresponding post-processing software installed in our MR scanner. Then, all the functional MR images were sent to the PACS (Picture Archiving and Communication System) system in our working computers. The freehand ROIs were drawn along the margin of the rectal tumor on each slice consecutively by covering the whole rectal lesion. The delineation of ROIs on the PDFF and R2* maps was based on T2WI and DWI as references, and the regions with intra-luminal gas, necrosis, cystic change and bleeding should be eschewed. 

The above MR data were measured by two radiologists specializing in abdominal radiology with five and eleven years’ experience. They independently assessed all the MR images and delineated ROIs without knowing the clinicopathological information of patients. The final values of IDEAL-IQ and APTWI-derived parameters were the averaged values of all the measurements on all slices. 

### 2.4. Histopathological Analysis

Two gastrointestinal pathologists with six and thirteen years of experience, independently evaluated all the histological examinations. All the specimens, removed at surgical resection, were fixed with 10% formalin, cut into 4-μm sections, and stained with hematoxylin and eosin (H.E.). The pathological characteristics of rectal cancer were as follows: tumor invasion of rectal wall (T stage), lymph node involvement (N stage), histological grade, status of mesorectal fascia (MRF) invasion and extramural venous invasion (EMVI).

With regard to the gland/tubule formation and tissue structure in rectal tumors, the histological tumor grade was categorized into G1 (well-differentiated tumor with over 95% gland formation), G2 (moderately differentiated tumor with 50–95% gland formation) and G3 (poorly differentiated tumor with 0–49% gland formation). G1 and G2 tumors belonged to low-grade tumors and G3 tumors were classified as high-grade tumors, based on the WHO criteria for tumor grading system. 

A positive MRF status indicates that tumors/malignant lymph nodes are very near to the mesorectal fascia—within 1 mm of distance. Positive EMVI status refers to the finding of tumor components within an extramural endothelium-lined space, which is surrounded by a rim of muscle structure or contains red blood cells [27]. 

### 2.5. Statistical Analysis 

The Kolmogorov–Smirnov test was utilized to test the quantitative parameters of mean PDFF, R2* and MTRasym (3.5 ppm) for normal distribution. The independent sample t test or Mann–Whitney U test was performed to assess the differences of each parameter in relation to different status of histopathological features of rectal cancer. The different status of histopathological features refers to: pT1-2 versus pT3-4, pN0 versus pN 1-2, low grade (G1-2) versus high grade (G3), negative versus positive status of MRF and EMVI. The association of MRI parameters with histopathological features was analyzed by the Spearman correlation coefficient, which was also utilized for evaluating the relationship between PDFF/R2* and MTRasym (3.5 ppm). 

The receiver operating characteristic (ROC) curve was performed to assess the diagnostic performances of these MR parameters for prediction of different status of histopathological features of rectal cancer. The differences in the area under the ROC curve (AUC) of each parameter for assessment of histopathological features were compared using the Delong test. The parameters including the AUC, sensitivity, specificity, and Youden index were derived from the ROC curve analysis. 

The intra-class correlation coefficient (ICC) was also performed to analyze the interobserver repeatability between two radiologists on measurements of IDEAL-IQ and APTWI derived parameters. The statistical data analyses were performed using SPSS 19.0 (IBM, Armonk, NY, USA) and MedCalc 11.4.2 softwares (Mariakerke, Belgium). *p* values of <0.05 were considered statistically significant.

## 3. Results

### 3.1. Clinic and Pathologic Characteristics of Studied Population

The study cohort comprised 67 patients (42 males, 25 females, age range: 30–82 years, median: 58 years). There were 15, 22 and 30 patients with rectal cancer located at the upper, middle and lower segments of the rectum according to the location of rectal tumors, respectively. The IDEAL-IQ and APTWI parameters of different histopathological factors of rectal cancer were averaged in Table 2. The characteristics of all patients were displayed in Table 2.

### 3.2. Inter-Observer Variability for Assessment of IDEAL-IQ and APT Imaging-Derived Parameters

**Figure 2:**
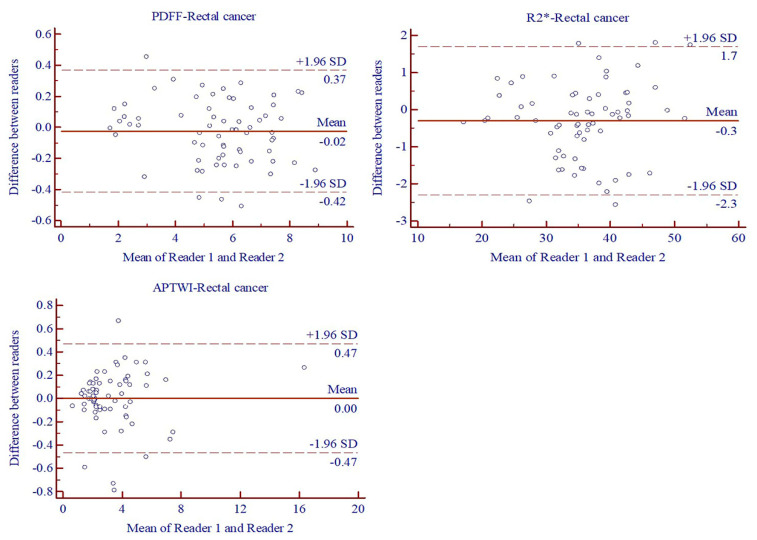
Bland–Altman analysis of PDFF, R2* and MTRasym (3.5 ppm) of patients with rectal cancer by two readers.

Excellent agreement was noted in the ICCs between two readers for measurement of quantitative parameters including PDFF (0.9968, 95% confidence interval (CI): 0.9948–0.9981), R2* (0.9952, 95%CI: 0.9922–0.9970) and MTRasym (3.5 ppm) (0.9970, 95%CI: 0.9952–0.9982). Moreover, the Bland–Altman statistics also demonstrated repeatable and reproducible measurements between two readers (Figure 2). 

### 3.3. Comparison of MR Parameters between Different Histopathological Features of Rectal Cancer

**Figure 3:**
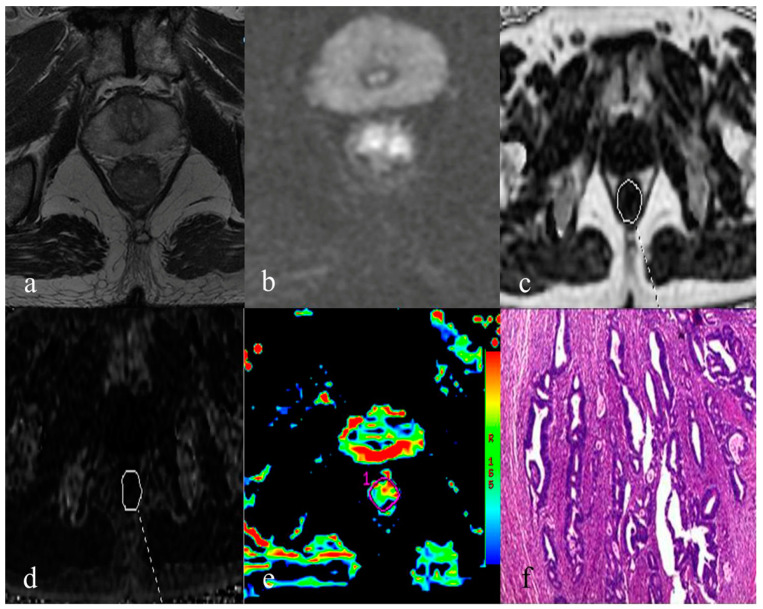
A 66-year-old male of rectal adenocarcinoma with T3 stage and no lymph node metastasis (moderately differentiated; EMVI-; MRF-). (**a**). axial T2-weighted imaging demonstrates a mass with moderate intensity in the rectum. (**b**). axial diffusion-weighted imaging indicates a mass with uneven high signal in the rectal lumen. (**c**). PDFF map with delineation of corresponding rectal tumor (PDFF = 3.60%). (**d**). R2* map with delineation of corresponding rectal tumor (R2* = 32.20 Hz). (**e**). pseudo-color map of APTWI indicates a mean MTRasym (3.5 ppm) of 3.56%. (**f**). Histopathological result indicates that the tumor invades the surrounding adipose tissue of rectum.

**Figure 4:**
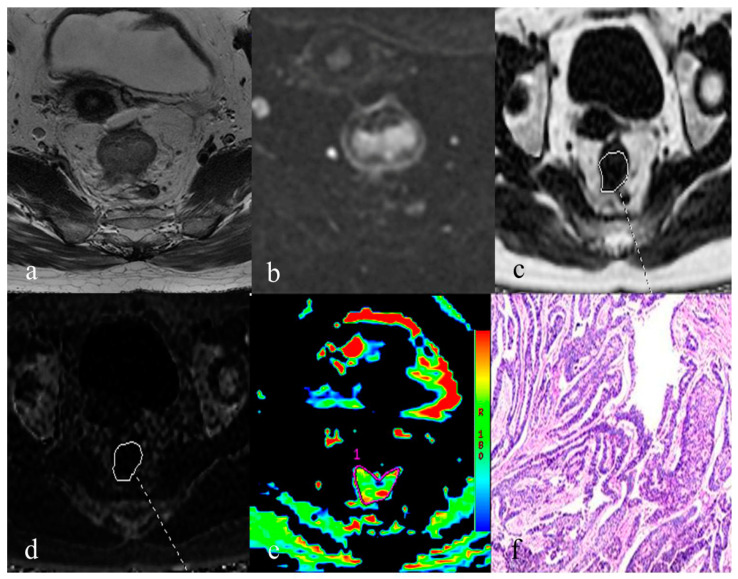
A 49-year-old female of rectal adenocarcinoma with T3 stage and N2 lymph node metastasis (moderately differentiated; EMVI+; MRF−). (**a**). axial T2-weighted imaging demonstrates an irregular-shape mass with moderate intensity in the rectum. (**b**). axial diffusion-weighted imaging indicates a mass with mildly high signal in the rectal lumen. (**c**). PDFF map with delineation of corresponding rectal tumor (PDFF = 4.51%). (**d**). R2* map with delineation of corresponding rectal tumor (R2* = 32.41 Hz). (**e**). pseudo-color map of APTWI indicates a mean MTRasym (3.5 ppm) of 4.17%. (**f**). Histopathological result indicates that the tumor invades the surrounding adipose tissue of rectum, and locally breaks through the serosal layer of rectum.

Significantly higher PDFF, R2* and MTRasym (3.5 ppm) were noted in the group with T3+T4 stage, metastatic lymph node, higher tumor grade, positive status of MRF and EMVI, than that with T1+T2 stage, non-metastatic lymph node, lower tumor grade, negative status of MRF and EMVI (*p* < 0.001 for PDFF, *p* = 0.000–0.015 for R2* and *p* = 0.000–0.006 for MTRasym (3.5 ppm)) (Table 2, Figure 3 and Figure 4). 

### 3.4. Association of PDFF, R2* and MTRasym (3.5 ppm) Parameters with T/N Stage, Tumor Grade, MRF and EMVI Status of Rectal Cancer

PDFF, R2* and MTRasym (3.5 ppm) correlated positively with different histopathological features of rectal cancer. The ranges of correlation degree (r) were 0.464–0.723 for PDFF (*p* < 0.001), 0.299–0.651 for R2* (*p* = 0.000–0.014), and 0.337–0.667 for MTRasym (3.5 ppm) (*p* = 0.000–0.005) (Table 3). 

### 3.5. Correlation of IDEAL-IQ Derived Parameters with MTRasym (3.5 ppm) from APTWI

**Figure 5:**
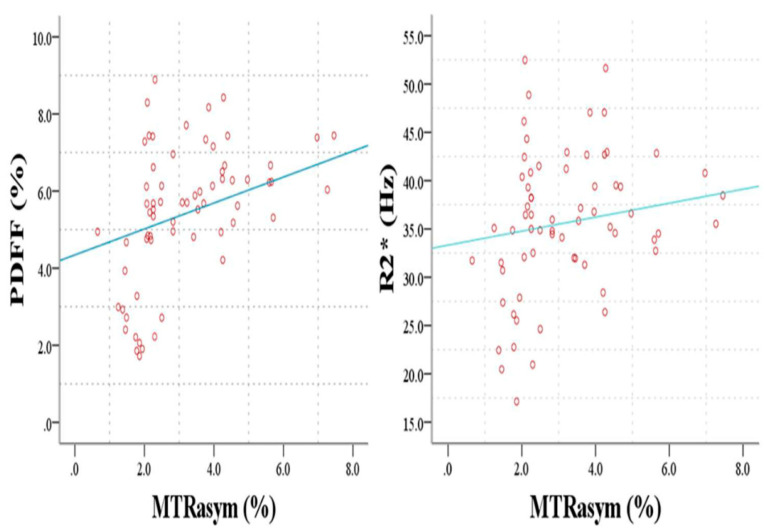
Correlation of MTRasym (3.5 ppm) with PDFF (r = 0.563, *p* < 0.001) and R2* (r = 0.335, *p* = 0.006).

There was a moderately positive correlation between MTRasym (3.5 ppm) and PDFF (r = 0.563, *p* < 0.001). Meanwhile, MTRasym (3.5 ppm) was also mildly correlated with R2* (r = 0.335, *p* = 0.006) (Table 4, Figure 5).

### 3.6. ROC Curve Analysis

**Figure 6:**
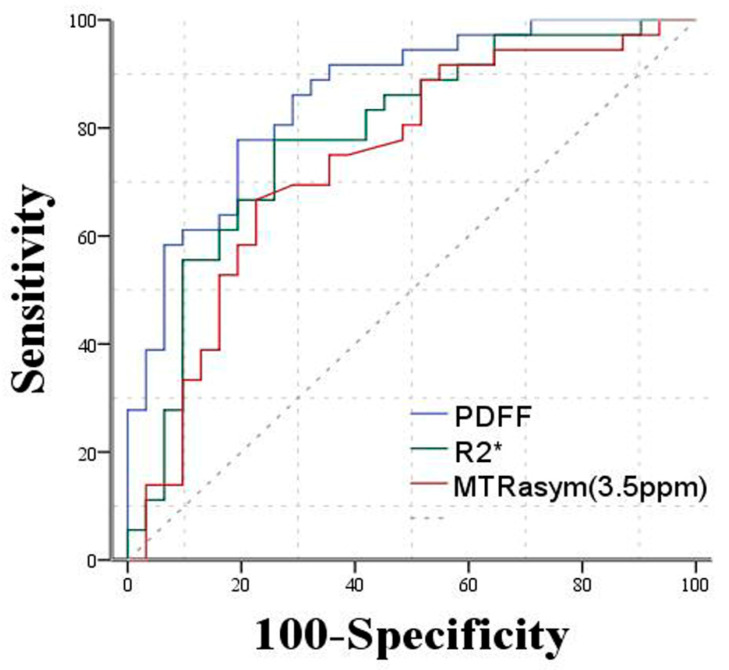
ROC curves of PDFF, R2* and MTRasym (3.5 ppm) for distinguishing metastatic/non-metastatic lymph nodes. ROC analysis demonstrated PDFF had a higher area under the curve (AUC = 0.858) than the other parameters.

Table 5 demonstrated the diagnostic performance of PDFF, R2* and MTRasym (3.5 ppm) for discrimination of histopathological features of rectal cancer, with the AUC ranges of 0.789–0.971 for PDFF (*p* < 0.001), 0.686–0.924 for R2* (*p* = 0.000–0.015) and 0.705–0.895 for MTRasym (3.5 ppm) (*p* = 0.000–0.006). The AUC of PDFF was significantly higher than that of MTRasym (3.5 ppm) for distinguishing metastatic from non-metastatic lymph nodes (z = 2.407, *p* = 0.0161). No significant difference was noted in the comparison of AUC of R2* and MTRasym (3.5 ppm) for nodal involvement (*p* > 0.05). As for other histopathological features of rectal cancer, there was no significant difference between these MR parameters (*p* > 0.05) (Figure 6). 

## 4. Discussion

In our study, we performed IDEAL-IQ and APTWI on patients with rectal cancer, and obtained the intra-tumoral fat fraction (PDFF), R2* and MTRasym (3.5 ppm) from tumor regions. Regarding the available MR data, we found that these MR parameters were associated with histopathological features of rectal cancer, such as T/N stage, histological differentiation, MRF and EMVI. The AUC of PDFF was significantly greater than that of MTRasym (3.5 ppm) in distinguishing metastatic from non-metastatic lymph nodes, but no other statistical differences were noted between IDEAL-IQ and APTWI parameters in prediction of the other histopathological features of rectal cancer. The IDEAL-IQ and APTWI are advantageous in imaging adipose tissue and tumor protein, and both functional techniques could be applied to clinical workflow by combining with immunohistochemical analysis in the future radiology diagnosis, although IDEAL-IQ and APTWI techniques need to be optimized in both image quality and scanning parameters.

We demonstrated that higher PDFF and R2* values were found in rectal tumors with higher T stage, lymph node metastasis, higher tumor grade, and positive status of MRF and EMVI. Adipose tissue is an endocrine organ, which could secrete cytokines and adipokines by adipocytes [28,29]. These cytokines and adipokines might finally promote inflammation and tumorigenesis of rectal cancer [30]. The adipose tissue microenvironment is closely associated with tumor formation and progression because of adipokines and fatty acids provided to tumor cells [31]. Moreover, accompanied angiogenesis also plays a key role in aggravation of histopathological features of rectal cancer with poor prognosis. Therefore, based on the findings from our investigation, it was assumed that intra-tumoral fat composition might potentially be related to the evolution process of tumor growth and prognosis of rectal cancer.

As for R2* parameter derived from the IDEAL-IQ sequence, there was a positive correlation between R2* and histopathological features of rectal cancer with advanced T/N stage, higher tumor grade, and positive status of MRF and EMVI. The R2* parameter was previously reported to quantify tumor hypoxia in animal and human studies [13,32,33]. Previous investigation indicated the increased blood deoxyhemoglobin content could result in elevated R2* [16]. The advanced stage tumors are manifested by gland formation, conspicuous cell atypia in tumor cells, nuclear polymorphism and increased tumor cell density of rectal cancer, which is depleted of oxygen in the tumor microenvironment by tumor consumption [34,35], accompanied by an increased level of deoxyhemoglobin concentration and R2* values. Moreover, tumor hypoxia is often accompanied by angiogenesis, which further aggravates the hypoxic status of tumor microenvironment and facilitates tumor progression and metastasis [36]. So the R2* parameter could be a potential biomarker for discrimination of the different status of T/N stage, tumor grade, MRF and EMVI of rectal cancer.

With regards to APTWI, significantly higher values of MTRasym (3.5 ppm) were found in rectal cancer with higher T stage, nodal involvement, higher tumor grade and positive status of MRF/EMVI. APTWI is a relatively novel molecular MR sequence, which could detect tumor proteins and peptides. APTWI is founded on the chemical exchange saturation transfer process and the signal intensity of APTWI is produced from the exchange of protons between endogenous protein/peptides and bulky water. Rectal cancer with the above histopathological features shows biological behaviors with aggressive manners, and is often featured by large tumor size, increased tumor cells/cellular density and excessive cell proliferation rate [37]. High-level tumor cell proliferation necessitates growing protein synthesis, leading to abnormal accumulation of peptides and proteins in the local tumor microenvironment. Hence, an augmented tumor proliferation pattern and tumor-related protein synthesis are the primary reasons for the enhanced APT signal (high MTRasym (3.5 ppm) value) by APTWI. Besides, tumor angiogenesis was reported to be another important factor, which could influence the APT signal and increase the MTRasym (3.5 ppm) value in rectal cancer with higher T/N stage, poor histological differentiation and positive status of MRF/EMVI status [19]. Abnormal vessel hyperplasia can often be present in rectal cancer with EMVI positive status, and increased vasculature permeability/blood perfusion are noted, which could be the potential reason for high APTWI signal. Based on the above findings, we hypothesize that APTWI could image protein synthesis by tumor cell proliferation, and that protein-associated angiogenesis in the tumor microenvironment is closely intertwined with a high APT signal.

More interestingly, we found that PDFF and R2* were moderately and mildly correlated to MTRasym (3.5 ppm), respectively. Previous studies [38,39] indicated that adipose tissue and hypoxia could help facilitate tumor growth. This result of association of PDFF and R2* with MTRasym (3.5 ppm) was consistent with previous findings. This finding indirectly implied that intra-tumoral fat tissue (PDFF) and hypoxia (R2*) might promote tumor cell proliferation, indicated by MTRasym (3.5 ppm) from APTWI. However, the above results were found based on the MR data from the rectal cancer patients collected from our hospital with a limited study number, so more patients should be enrolled to test the repeatability and validity of our findings.

We also evaluated the diagnostic performance of IDEAL-IQ and APTWI in analysis of histological features of rectal cancer. PDFF was significantly superior over MTRasym (3.5 ppm) for distinguishing metastatic from non-metastatic lymph nodes from our ROC analysis. No statistical differences between the diagnostic abilities of PDFF, R2* and MTRasym (3.5 ppm) were found in discriminating histological features of rectal cancer. The above results might be due to the following reasons: (1) it was under exploration for finding factors for causing variations of peptides and proteins in the tumor microenvironment; (2) the APTWI still needs improvement for scanning parameters, slices and scanning time; (3) APTWI could be influenced by the T1 relaxation time, local environment temperature and tissue pH, and the measurement of the parameter from APTWI might potentially be affected by the above factors.

Several limitations are present in our study. First, our sample size of studied patients was limited, and more patients should be enrolled in future investigations; second, the scanning time for APTWI was too long, and the long scanning time might cause potential occurrence of artifacts; third, the homogeneity of regional MR signal intensity is essential for APT imaging of the rectum, and intra-luminal gas existence and bowel movement would lead to uneven MR signal intensity and influence the APT signal, even though racanisodamine hydrochloride was utilized to reduce bowel movement. Fourth, only patients with rectal cancer were chosen for IDEAL-IQ and APT-weighted imaging, and other histological types of rectal tumors should be taken into consideration in future studies. Finally, the publicly available databases of IDEAL-IQ/APTWI-derived parameters and associated histopathological factors of rectal cancer did not exist, thus limiting the generalizability of our findings. 

In conclusion, IDEAL-IQ and APTWI are proved to be applicable to imaging rectal cancer based on our investigation. Both sequences could be used as noninvasive modalities for discrimination of histopathological features of rectal cancer. 

## Figures and Tables

**Table 1:** MR imaging protocol parameters.

Parameters	T1-Weighted Imaging	T2-Weighted Imaging	DWI	IDEAL-IQ	APTWI
Sequence	FSE	FSE	EPI	GRE	EPI
Orientation	Axial	Axial	Axial	Axial	Axial
TR/TE (msec)	500/11	5629/85	4000/75	5.9/2.6	4070/20
FOV (mm^2^)	380 × 380	200 × 200	200 × 100	380 × 380	360 × 360
Matrix	320 × 224	448 × 314	128 × 64	160 × 160	128 × 128
Slice thickness (mm)	3	2	3	3.5	4
Flip angle (degree)	111°	111°	N/A	3°	N/A
Bandwidth (Hz)	62.5	31.3	250	111.1	250
b-values (s/ mm^2^)	N/A	N/A	0, 800	N/A	N/A
NEX	2	4	12	0.5	1
Scan time (min:s)	1:44	4:04	2:32	0:19	4:29

FSE: fast spin echo; EPI: echo planar imaging; GRE: gradient recalled echo; TR/TE: repetition time/echo time; FOV: field-of-view; NEX: number of excitations.

**Table 2:** IDEAL-IQ and APT parameters of different histopathologic features of rectal cancer.

	PDFF (%)	*p*-Value	R2* (Hz)	*p*-Value	MTRasym (%)	*p*-Value
pT category						
T1+T2 (n = 18)	3.18 ± 1.23	<0.001	27.72 ± 5.95	<0.001	1.91 ± 0.79	<0.001
T3+T4 (n = 49)	6.32 ± 1.06		38.71 ± 5.33		3.89 ± 2.32	
pN category						
N0 (n = 31)	4.33 ± 1.65	<0.001	32.09 ± 7.22	<0.001	2.92 ± 2.74	0.001
N1+N2 (n = 36)	6.46 ± 1.21		38.92 ± 5.88		3.74 ± 1.55	
Tumor grade						
G1+G2 (n = 44)	4.83 ± 1.70	<0.001	33.63 ± 7.15	0.002	2.95 ± 1.65	0.006
G3 (n = 23)	6.71 ± 1.19		39.83 ± 5.93		4.15 ± 2.87	
MRF						
Negative (n = 46)	4.95 ± 1.81	<0.001	34.29 ± 7.76	0.015	2.92 ± 2.43	<0.001
Positive (n = 21)	6.62 ± 1.04		38.97 ± 5.15		4.20 ± 1.27	
EMVI						
Negative (n = 37)	4.55 ± 1.68	<0.001	33.01 ± 7.48	0.001	2.30 ± 0.80	<0.001
Positive (n = 30)	6.62 ± 1.22		39.15 ± 5.61		4.67 ± 2.66	

Data are means ± standard deviations. MRF: mesorectal fascia; EMVI: extramural vascular invasion.

**Table 3:** Association of PDFF, R2* and MTRasym (3.5 ppm) parameters with T/N stage, tumor grade, MRF and EMVI status of rectal cancer.

Histological Features	PDFF (%)		R2* (Hz)		MTRasym (%)	
	r-Value	*p*-Value	r-Value	*p*-Value	r-Value	*p*-Value
pT category	0.723	<0.001	0.651	<0.001	0.606	<0.001
pN category	0.619	<0.001	0.492	<0.001	0.413	0.001
Tumor grade	0.507	<0.001	0.385	0.001	0.337	0.005
MRF	0.464	<0.001	0.299	0.014	0.524	<0.001
EMVI	0.607	<0.001	0.427	<0.001	0.667	<0.001

MRF: mesorectal fascia; EMVI: extramural vascular invasion.

**Table 4:** Association of PDFF and R2* with MTRasym in assessment of rectal cancer.

Parameter	PDFF (%)		R2* (Hz)	
	r-Value	*p*-Value	R-Value	*p*-Value
MTRasym (%)	0.563	<0.001	0.335	0.006

**Table 5:** ROC analysis of the diagnostic performance of different parameters in distinguishing histological features of rectal cancer.

Parameters	AUC (95% CI)	Cutoff	Sensitivity (%)	Specificity (%)	Youden Index	*p*-Value
pT category						
PDFF	0.971 (0.928–1.000)	4.95	89.80	94.40	0.842	<0.001
R2* MTRasym	0.924 (0.849–0.999) 0.895 (0.799–0.990)	31.85 1.98	95.90 100.00	77.80 72.20	0.737 0.722	<0.001 <0.001
pN category						
PDFF	0.858 (0.770–0.946)	5.68	77.80	80.60	0.584	<0.001
R2* MTRasym	0.785 (0.672–0.898) 0.739 (0.616–0.861)	35.15 2.96	77.80 66.70	74.20 77.40	0.520 0.441	<0.001 0.001
Tumor grade						
PDFF	0.808 (0.702–0.915)	6.31	65.20	88.60	0.539	<0.001
R2* MTRasym	0.734 (0.611–0.857) 0.705 (0.581–0.828)	38.88 2.30	60.90 82.60	81.80 56.80	0.427 0.394	0.002 0.006
MRF						
PDFF	0.789 (0.682–0.895)	5.57	90.50	60.90	0.513	<0.001
R2* MTRasym	0.686 (0.558–0.814) 0.826 (0.725–0.927)	32.30 3.65	95.20 76.20	39.10 82.60	0.344 0.588	0.015 <0.001
EMVI						
PDFF	0.852 (0.762–0.942)	6.14	70.00	89.20	0.592	<0.001
R2* MTRasym	0.748 (0.630–0.865) 0.887 (0.808–0.967)	32.30 3.81	96.70 70.00	48.60 97.30	0.453 0.673	0.001 <0.001

MRF: mesorectal fascia; EMVI: extramural vascular invasion.

## Data Availability

The data associated with the findings of our investigation are available from the corresponding author (Z.L.) upon reasonable request.
